# Menstrual cycle tracking in professional volleyball athletes

**DOI:** 10.3389/fspor.2024.1408711

**Published:** 2024-06-28

**Authors:** Andrea Roffler, Marie-Therese Fleddermann, Hanna de Haan, Karsten Krüger, Karen Zentgraf

**Affiliations:** ^1^ Department of Movement Science and Training in Sports, Institute of Sport Sciences, Goethe University Frankfurt, Frankfurt am Main, Germany; ^2^ Department of Performance Psychology, German Sport University Cologne, Köln, Germany; ^3^ Department of Exercise Physiology and Sports Therapy, Institute of Sports Science, Justus-Liebig University Giessen, Giessen, Germany

**Keywords:** menstrual cycle, volleyball, well-being, elite athletes, symptoms

## Abstract

**Introduction:**

The menstrual cycle may affect well-being and physical performance of elite female athletes by interfering with the function of multiple physiological systems. The aim of this study was to characterize the symptoms of the menstrual cycle and their frequency in elite female volleyball players.

**Methods:**

Twenty professional female volleyball players were instructed to track their menstrual symptoms over the course of the first German national league season using the FitrWoman® tracking app. The app recorded the cycle length, duration, and intensity of the period as well as the occurrence and frequency of frequent cycle symptoms. The reported symptoms were then categorized into four categories (frequently, sometimes, rare, never) in order to create an individual Menstrual Symptom index (MSi) for each athlete.

**Results:**

The most frequently occurring symptoms among all players without hormonal contraception (non-HC; *n* = 15) were “stomach cramps” (*n* = 15), “sleep disturbances” (*n* = 11), and “tiredness” (*n* = 11). The average number of symptoms counted per cycle was 11.8 (±17.7) and the average calculated MSi within the team was 12.9 (±10.7) points for non-HC users. The HC players (*n* = 4) also regularly experienced symptoms such as “sleep disturbances” or “tendered breasts”. The most common symptoms “stomach cramps” and “disturbed sleep” occurred more frequently during menstruation, while symptoms such as “bloating”, “cravings” or “tendered breasts” did also peak before menstruation.

**Discussion:**

Menstrual cycle symptoms can be highly individual within a professional sports team. The calculation of the MSi seems to be a simple and accessible method to describe and overview the intensity and prevalence of symptoms in top female athletes in sports games.

## 1 Introduction

In the last few years, attention towards the menstrual cycle and how it possibly influences performance and well-being in elite female sports has rapidly increased (1). The menstrual cycle represents a biological rhythm, which primarily regulates female reproductive function and is traditionally divided into phases based on hormonal fluctuations (2). Mechanisms underlying hormonal fluctuations are also believed to be the cause of regularly appearing symptoms (3), often referred as menstrual cycle symptoms (MCS) (4). A systematic review by Taim and colleagues shows a high variation in prevalence of different symptoms among athletes. For example, “abdominal cramps” is reported by between 47.5% and 70.0% of athletes during the premenstrual and menstruation phases in different studies (5). MCS are believed to have a negative impact on athletes' perceived performance (6, 7). Antero et al. found a negative correlation between the presence of MCS and the perceived performance of elite athletes (6), while in another study involving Australian elite athletes, 50.0% reported a negative influence of menstrual cycle phases on their performance during training, which increased to 56.5% for competition days (8). To reduce these irregularities and symptoms during the menstrual cycle, hormonal contraceptives (HC) are often prescribed (9, 10). But also women using HC experience recurring symptoms, despite not having a natural menstrual cycle (6, 9, 10). Most athletes with a natural cycle report the highest prevalence and influence of symptoms during the premenstrual and menstruation phases (3, 6, 8, 10). A higher prevalence of symptoms before menstruation can be associated with the premenstrual syndrome (PMS), which is characterized by a combination of physical and psychological symptoms before the onset of menstruation and during menstruation. These symptoms include “depressed mood”, “anxiety or tension”, “irritability”, and “lack of energy” among others (11). In a non-athlete population, prevalence of PMS in studies ranges from 32.6% to 62.9% and seems to be dependent on the country in which the study was performed (12). For an athlete population, Taim et al. show a prevalence of PMS between 8.6% and 59.6% across seven studies (5). The high variations in prevalence could also be due to the different definitions used for PMS (5). For professional athletes, PMS is more likely to influence performance compared to non-professional athletes (13). Another risk factor for reduced performance due to PMS is the presence of the symptoms “difficulty in concentration” or “fatigue/lack of energy” (13). Even though studies report a decreased perceived performance mostly in the premenstrual or menstruation phase (6, 8), objective data regarding performance fluctuations during menstrual cycle remains equivocal (14). Nevertheless, MCS are an important factor in athletes' well-being and perceived performance. Tracking menstrual cycle could help coaches access important information about their athletes if desired. However, integrating menstrual cycle considerations into practice and lifestyle can be challenging. Alongside to limitations in resources and knowledge, there is a high degree of intra- and inter-individual variability in menstrual cycle parameters. Adapting strategies can be even more challenging considering menstrual cycle disturbances which are more prevalent in elite athletes (5, 15, 16). Athletes in general have a higher risk of experiencing anovulatory cycles and menstrual disturbances, including oligomenorrhea, luteal phase defects, primary and secondary amenorrhea among others (5, 17, 18). This could be due to high levels of stress and excessive exercise in combination with restricted diet (19, 20), leading to a low energy availability (LEA) (21). Further research is needed to explore effective strategies for prevention and management, including communication with coaches and other team members, since many athletes report a lack of structured communication, fundamental knowledge, and the feeling that their coaches would not understand them when talking about menstrual cycle (22–25). Hence, menstrual cycle still appears to be a taboo topic. While menstrual cycle tracking provides valuable insights, its utility may be limited due to the necessity of accurately determining menstrual phases, which requires consideration of hormonal fluctuations and other physiological factors (26, 27). This is not always feasible in training environment due to financial and methodological constraints. Julian and Sargent suggest that using both menstruation diaries and well-being tracking measures together may provide a useful tool for predicting individual athletes' menstrual phases and cycle duration in a training environment while minimizing individual effort (16). Bruinvels et al. introduced a method to quantify prevalence and occurrence of symptoms in a single value called Menstrual Symptom index (MSi) which includes both frequency and number of symptoms (4). This measure could help to assess the impact of MCS in a sports team and to quickly screen a team for potential need for interventions (4). There is still a notable gap in available data concerning the variability of symptoms and comprehensive tracking information among an entire professional female sports team. Therefore, the present study aimed to examine the monthly recurring symptoms of an elite female volleyball team over four to twenty months via, among others, the MSi introduced by Bruinvels et al. (4).

## 2 Materials and methods

### 2.1 Study design

An observational design was used to obtain an overview of the variety of MCS of an elite female volleyball team. Data on the timing of menstrual bleeding and occurring symptoms were collected during the 2019/2020 and 2020/2021 competitive seasons. Players using HC also reported symptoms and their data regarding withdrawal bleeding as, even though they do not have a natural cycle, we expected them not to be free from recurring symptoms (6, 9, 10).

### 2.2 Participants

Twenty elite female volleyball athletes between 19.2 and 27.7 years (age 24.5 ± 2.0 years; BMI 22.8 ± 1.6 kg/m^2^) from the first German national league participated in the study. Not all athletes were tracked for the same period due to changes in the team setup, e.g., when the player's club contract ended. For example, athlete VB05 was tracked for 20 months whereas athlete VB10 was tracked for nine months. The exact participation durations and the corresponding numbers of cycles between two bleedings can be found in Table 1. One athlete (VB18) was excluded from all symptom-based descriptions because she did not report at least one complete cycle during her measurement period.

**Table 1:** Participants’ demographic and menstrual cycle characteristics.

Athlete	Measurement span (months)	Number of full cycles measured	Contraception	Cycle length (days)	Bleeding length (days)	Symptoms per cycle	MSi	Symptoms occurring often
VB01	16.5	17	Vaginal ring	28.0 ± 1.1	3.5 ± 0.9	3.5 ± 2.2	8	0
VB02	16	17		27.7 ± 11.4	4.7 ± 1.3	0.3 ± 0.6	2	0
VB03	16.5	18	Hormone IUD	27.7 ± 1.8	4.6 ± 1.1	1.9 ± 3.5	9	0
VB04	16.5	18		27.6 ± 2.5	6.8 ± 1.7	14 ± 9.1	22	4
VB05	20	18		31.6 ± 2.9	5.6 ± 1.8	1.5 ± 2.2	5	0
VB06	16	16		29.8 ± 7.3	2.8 ± 1.5	0.1 ± 0.3	1	0
VB07	17	9		53.9 ± 46.7	3.6 ± 0.7	1.0 ± 1.6	8	0
VB08	11	6	OC	53.3 ± 25.7	4.0 ± 1.4	6.7 ± 10.1	9	0
VB09	9.5	9		32.4 ± 13.0	4.9 ± 2.5	6.6 ± 5.8	16	0
VB10	9	10		26.6 ± 2.4	5.0 ± 1.1	2.9 ± 3.2	6	0
VB11	9	6	OC	45.0 ± 22.9	9.8 ± 5.8	10.0 ± 16.2	18	0
VB12	8	7		30.4 ± 1.3	4.8 ± 0.7	7.6 ± 6.8	15	0
VB13	8	8		27.6 ± 6.0	3.9 ± 0.9	28.0 ± 16.4	19	2
VB14	8	5		45.0 ± 33.6	4.7 ± 1.6	1.4 ± 2.0	4	0
VB15	7	3		64.7 ± 21.6	6.3 ± 0.5	61.0 ± 13.1	34	8
VB16	4	3		23.0 ± 6.0	6.0 ± 1.4	39.7 ± 8.5	33	8
VB17	4	2		27.0 ± 2.8	2.3 ± 1.2	1.5 ± 2.1	4	0
VB18	4	0		n/a	1.5 ± 0.7	1.0 ± 0.0	n/a	0
VB19	4	3		26.7 ± 1.2	3.3 ± 1.3	3.7 ± 1.5	6	0
VB20	4	2		35.5 ± 10.6	3.3 ± 2.1	8.0 ± 5.7	18	2
Mean score (M)	10.1	8.4		34.9 ± 11.6	4.6 ± 1.8	10.0 ± 15.6	12.5 ± 9.7	1.1

Team demographics and menstrual cycle characteristics of each female athlete. Cycle length in months, period length, and symptoms per cycle are all shown as mean values with standard deviation. Bleeding length refers to both menstrual and withdrawal phase bleeding. HC, hormonal contraception; MSi, menstrual symptom index; OC, oral contraceptives/contraception; IUD, intra-uterine device.

Moreover, four athletes were using different HC. VB01 was using a vaginal ring, VB03 used a hormonal intra-uterine device (IUD), while VB08 and VB11 were both using a combined oral contraceptive pill (Maxime and Cedia20, respectively). Although HC users do not have a biological menstruation, all athletes were asked to report recurring symptoms and data regarding the withdrawal bleeding in order to explore the scope of symptoms in a representative athlete population.

The players gave their informed consent before they participated in the study. The study was conducted in accordance with the Declaration of Helsinki and the protocol was approved by the local Ethics Committee (2021‒30, 28 June 2021).

### 2.3 Tracking tool

The data was assembled through an application called FitrWoman® (28), which has been used in previous studies to track the menstrual cycle (4, 24, 29). The app includes a calendar that gives an overview of the previous and current cycle phase. Below the calendar view, athletes can directly log bleeding intensities (including none, spotting, light, medium, heavy). Moreover, the athletes can track their symptoms in the app by choosing symptoms out of a selection of eighteen MCS (“stomach cramps”, “fatigue”, “bloating”, “muscle ache”, “heavy legs”, “disturbed sleep”, “cravings”, “tendered breasts”, “headache”, “diarrhea”, “stressed”, “irritability”, “weak”, “high temperature”, “poor concentration”, “constipation”, “increased breathing”, and “nausea”). In addition, there is also a “notes” section to track symptoms that are not listed within the app or to write comments about special events. Participants were instructed to fill out the report every evening before sleeping, providing information on the occurrence of every symptom experienced—independently of the menstruation phase—as well as data regarding menstruation and for athletes using HC reporting data with respect to withdrawal bleeding.

By tracking the exact days of menstruation of each athlete for at least four months, the approximation of the participant's individual cycle occurrence were calculated (30, 31). “Phase 1” represents the duration of the menstruation bleeding and is, therefore, called “menstruation”. “Phase 2” lasts from the end of menstruation until the individual's middle of the cycle (calculated ovulation) and is labeled “follicular phase”. “Phase 3” counts the days from the middle point of the individual cycle to the start of the next menstrual bleeding, representing “luteal phase”. In order to classify symptoms further, in this study a fourth phase (“late luteal phase”) was additionally calculated, referring to the last five days of a cycle similarly to Solli et al. (3). As athletes using HC do not experience a natural menstrual cycle, no menstrual phases were calculated for this group.

### 2.4 Menstrual Symptom index (MSi)

The symptom data were classified according to a classification system suggested by Bruinvels et al. (4). The symptoms of every individual cycle were listed and categorized into four categories based on the frequency: “often” if the symptom appeared in every menstrual cycle (3 points), “sometimes” if the symptom appeared in every second cycle (2 points), “rare” if the symptom appeared in less than every second cycle (1 point), and “never” (0 points). The points of every symptom were summarized, resulting in a score ranging from zero points (all symptoms “never”) to a maximum score of 54 points (every symptom “often”). In this study, there were no additional symptoms which were entered manually through the comment section.

For athletes using HC, an equivalent calculation was performed based on data regarding withdrawal bleeding. The MSi has been developed for eumenorrheic athletes only and should therefore not be used for athletes on HC. Nevertheless, since current research shows, that HC users are not free from symptoms (6, 9, 10), in this study a similar calculation was performed for athletes on HC to describe all athletes of the team in a comparable manner.

### 2.5 Statistical analysis

All data is provided on the descriptive level showing mean values (*M*) with standard deviations (*SD*) calculated by using Microsoft Excel (Version 2211). Figures were created using GraphPad Prism (Version 10.1.0). Descriptive values regarding cycle data only includes athletes with a natural cycle, which means excluding VB01, VB03, VB08, and VB11. Additionally, we excluded VB18 for all calculations because no fully reported cycle was available.

## 3 Results

The athletes' characteristics are presented in Table 1. The counts of noticed symptoms per cycle ranged from 0.3 (±0.6) symptoms to 61.0 (±13.1) symptoms per cycle. On average, athletes reported 10.0 (±15.6) symptoms per cycle. The measured cycles varied in length among the athletes without HC from 64.7 (±21.6) days to the shortest cycle length of 23.0 (±6.0) days (Table 1). The longest menstruation length was 9.8 (±5.8) days. The shortest period length was 2.3 (±1.2) days.

**Figure 1:**
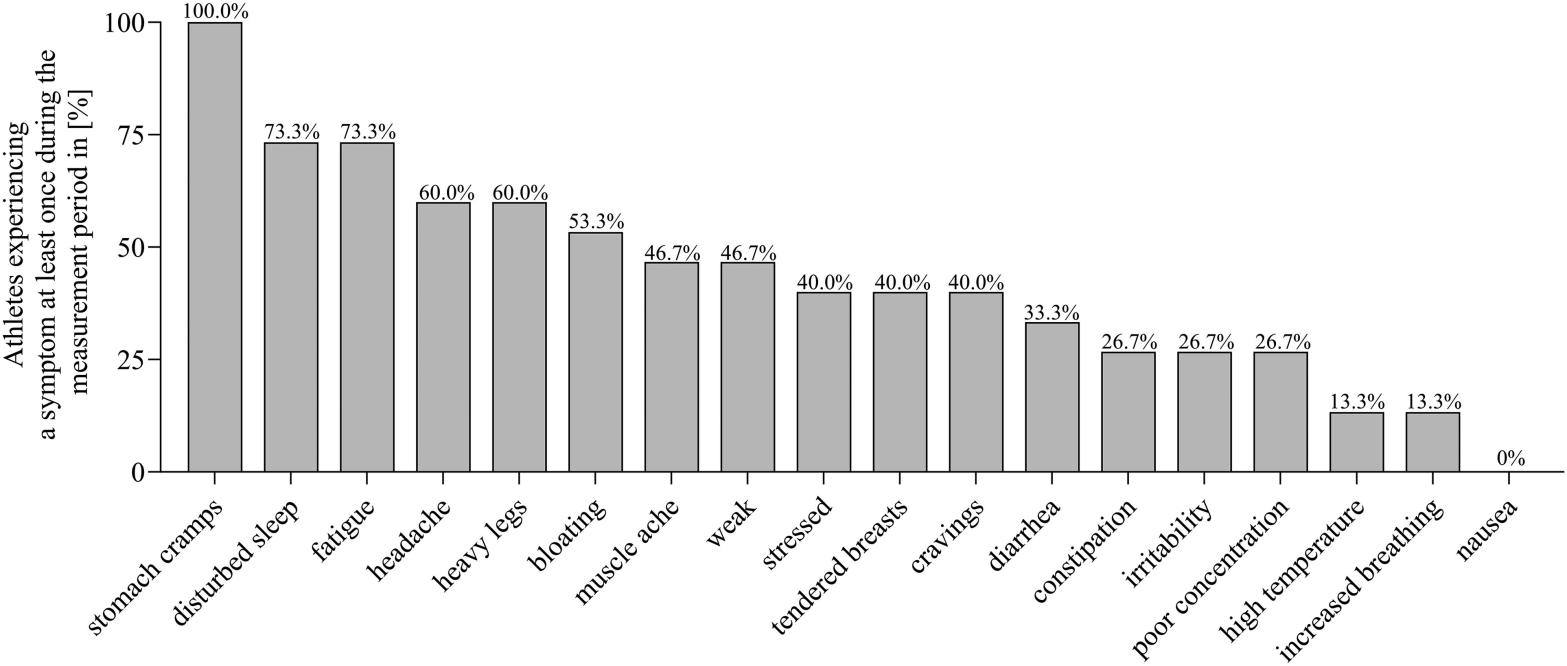
Prevalence of symptoms reported at least once during the individual measurement period among all athletes without hormonal contraception.

Figure 1 shows the percentage of athletes without HC experiencing the different symptoms for at least once during the measurement period. All athletes experienced “stomach cramps” at least once. “Disturbed sleep” and “fatigue” were reported by 73.3% of all athletes at least once. “Nausea” was the least frequently reported symptom, occurring in only one case.

**Figure 2:**
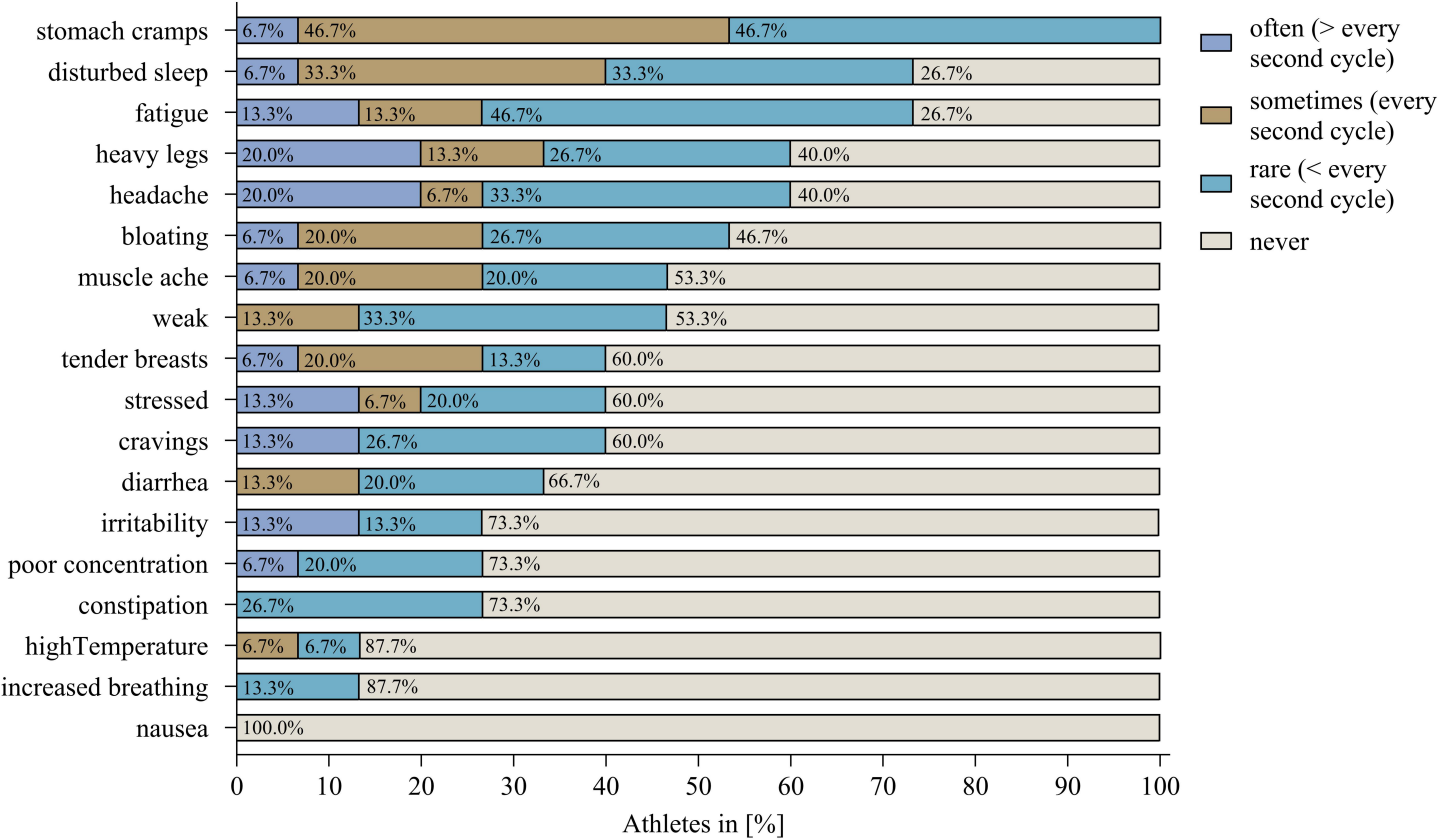
The percentage of athletes without hormonal contraception reporting the respective symptom in the frequency categories: “often”, “sometimes”, “rare”, and “never”.

Additional information is provided in Figure 2 which includes the frequency of the symptoms. The most common symptom, “stomach cramps”, was experienced “often” by only one athlete resulting in 5.3% of athletes. “Headache” and “heavy legs”, on the other hand, were experienced “often” together in three athletes. Also, all four HC users regularly reported symptoms. VB1 (vaginal ring), VB03 (hormonal coil), and VB08 (oral contraceptive) experienced “tendered breasts” the most, whereas VB11 (oral contraceptive) experienced “fatigue” regularly.

**Figure 3:**
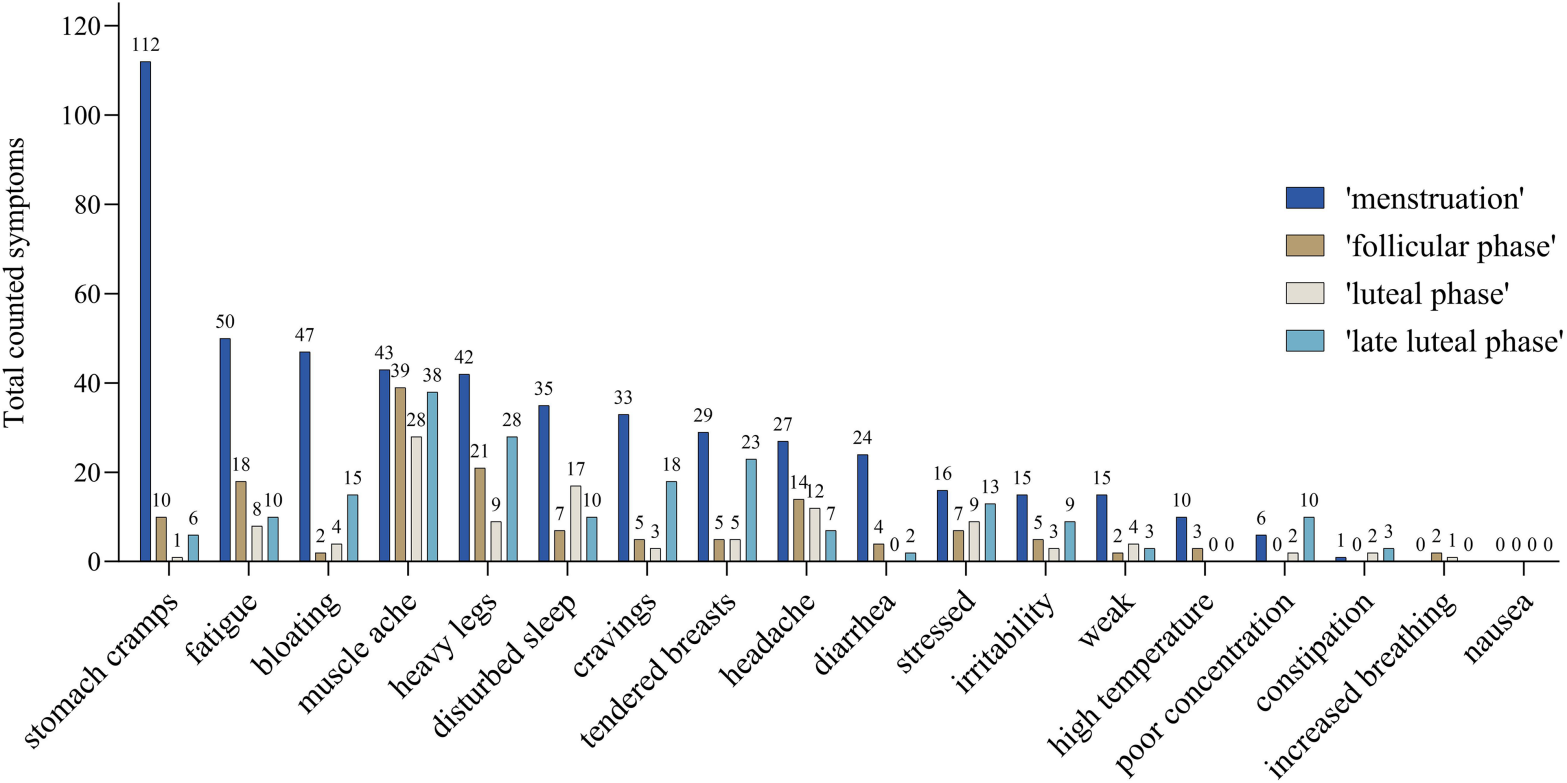
Number of counted symptoms of all athletes without hormonal contraception occurring in the different calculated cycle phases: “menstruation” (blue), “follicular phase” (brown), “luteal phase” (grey), and “late luteal phase” (light blue) which refers to the last five days of the cycle. The number, e.g., 47 of the symptom “bloating” refers to 47 reports during menstruation phase.

Total reports of symptoms were the highest during the “menstruation” (0.79 symptoms per day) followed by “late luteal phase”, “follicular phase”, and “luteal phase” (0.08, 0.09, and 0.00 symptoms per day, respectively). Figure 3 shows the absolute number of symptoms among the different cycle phases of “menstruation”, “follicular phase”, “early luteal phase”, and “late luteal phase”. When considering single symptoms, thirteen out of eighteen symptoms were more frequent during “menstruation”.

**Figure 4:**
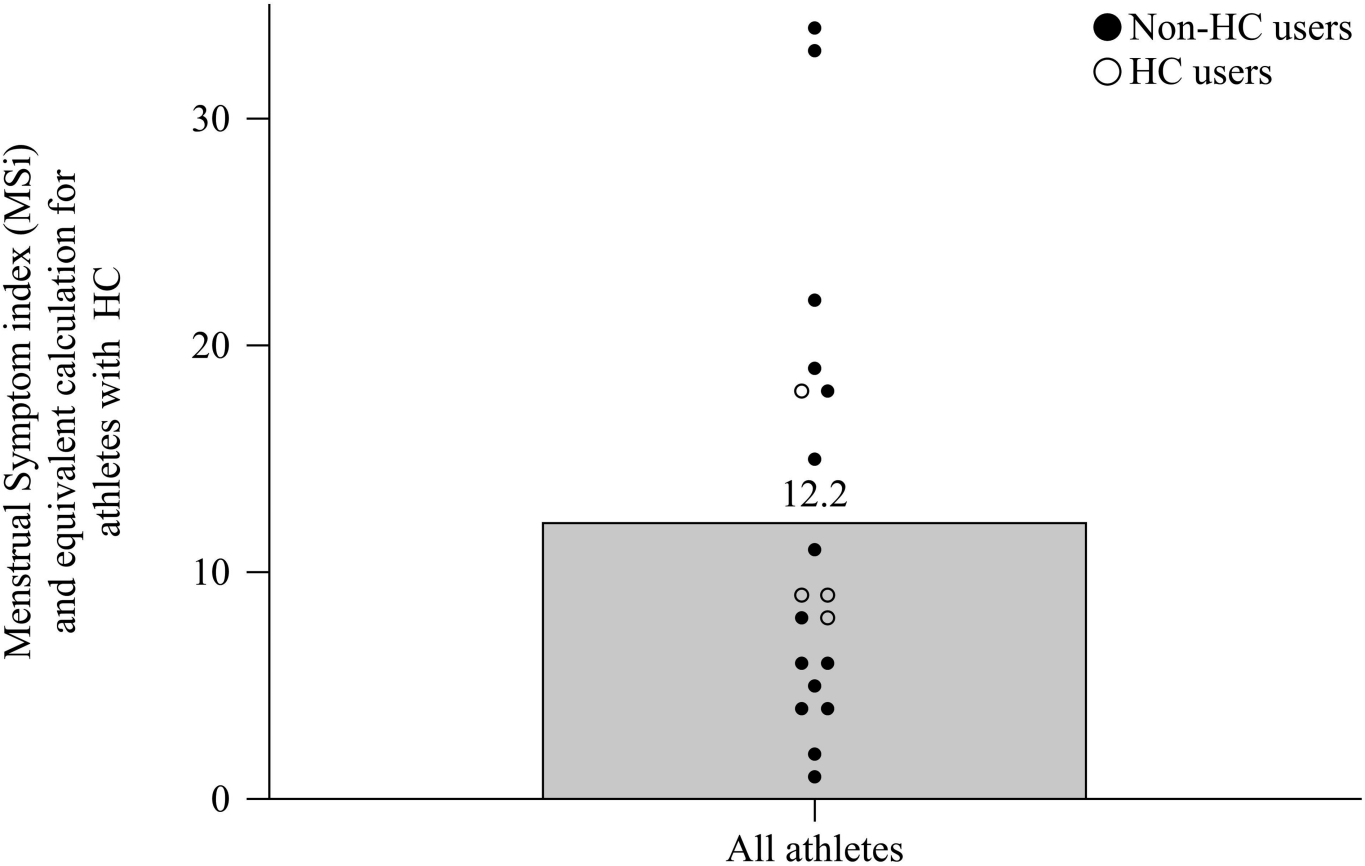
The MSi scores from all athletes without hormonal contraception and the equivalent calculation for athletes with hormonal contraception shown as mean and individual scores.

The mean number of reported symptoms per cycle was 11.8 (±17.7). The lowest MSi score was one point (VB06) and the highest MSi scores were 34 and 33 points (VB15 and VB16, respectively) out of a maximum of 54 points (every symptom often). Figure 4 shows the individual MSi scores for all athletes including non-HC users (mean score 12.5 ± 10.7) and the equivalent calculations for HC users (mean score 11.05 ± 4.7 points).

## 4 Discussion

The aim of the study was to describe an elite volleyball team by their recurring symptoms and their menstruation tracking data. Findings of this study illustrate the diversity of symptoms and menstrual cycle characteristics, e.g., cycle length, among an elite female volleyball team. Variations in symptom recurrence was shown for non-HC users as well as HC users. Out of 18 symptoms, 6 were reported by more than half of all non-HC athletes. The most commonly reported MCS among the team in this study were “stomach cramps”, “disturbed sleep”, “bloating”, “fatigue”, and “heavy legs”. “Stomach cramps” and “bloating” were predominantly tracked during the menstruation itself. A recent study from Kullik et al. investigates the symptom prevalence in active women and athletes in Germany (32). In their study, the most prevalent symptoms are “cravings/increased appetite”, “mood changes/anxiety”, and “tiredness/fatigue”. This could be because the study includes women with HC. The authors did find a small positive relationship between the use of HC and anxiety as well as with sleep behavior (32). Athletes using HC also experience regular symptoms such as “tendered breasts” and “fatigue”, which were both reported by all HC users at least once. This finding supports current research, that shows negative symptoms (e.g., “stomach cramps”, “back pain”, and “headaches”) in both non-HC users and HC users (6, 10, 33).

To overview symptom prevalence and frequency, a quantification method called MSi (minimum 0 points, maximum 54 points) was used. In this study the mean MSi was 12.5 points (±10.7), which is comparable with the mean MSi score from physical active women in the study of Bruinvels et al. (4) reporting 17.9 ± 10.1 points for active German women. A study of Kullik et al. reports a slightly higher MSi of 20.1 ± 10.2 points for active women including athletes in Germany (32). Differences could be due to the investigated population or due to differences in methodology. Kullik et al. (32) included both non-elite and elite athletes with and without HC. Athletes could differ from active women due to the higher load in training and exercise, which may have an impact on the menstrual cycle physiology (20). Additionally, the symptom definition was slightly adapted which could cause differences. Individual data of the MSi shows a high variation. One female athlete experienced a very low MSi score of one point. Nevertheless, there were also two athletes suffering from an MSi score of 33 respectively 34 points out of the maximum 54 points. When carrying out an equivalent calculation for HC users, all four athletes showed at least 8 points, which, again, supports current research (6, 9, 10), that HC users are not free from symptoms. However, due to the low number of athletes using HC in this study, the interpretation of the results is limited. Additionally, in this study there is no information available about the individual history of HC. Some athletes may have started HC to manage symptoms (9, 33). Martin et al. (34) investigated symptoms in both HC and non-HC users and concluded, that HC users are more likely to report positive effects in form of reduced symptoms after starting contraception. Furthermore, we do not know if some of the symptoms reported in this study might also be side-effects of the contraceptive use (4).

By finding a solutional approach for specific recurring symptoms, well-being of athletes might be improved. Around 58% of elite Australian female athletes feel that the menstrual cycle harms their performance (9). In addition, women with a higher number and frequency of symptoms are more likely to miss training or competition (4). By noticing regularities within one athlete regarding the time and occurrence of a symptom, it would be possible to adapt solutional approaches in daily life. For example, a reason for these abdominal symptoms could be that, in this phase, the endometrial lining is shed as menstrual bleeding resulting in discomfort (35). Tsai suggests that a yoga intervention could significantly reduce abdominal swelling, breast tenderness, abdominal cramps, and cold sweats (36). Another approach to minimize “stomach cramps” could be avoiding behaviors that increase inflammatory responses, for example, not drinking alcohol or minimizing the consumption of processed foods in the “late luteal phase”, as increased hs-CRP levels can cause premenstrual symptoms (37). By adapting nutrition or supplementing micronutrients, e.g., vitamin D or calcium, PMS might be reduced as well (38–42). In this regard, Farpour et al. demonstrate that PMS symptoms are more pronounced with a diet high in salt and sugar or a Western mixed diet (43). On the other hand, food such as vegetables and a low-fat, high-fiber diet can reduce the duration and intensity of PMS symptoms (37). A recent meta-analysis shows that omega-3 fatty acids were efficient in reducing the severity of PMS-symptoms (44).

Interestingly, studies show, that poor sleep quality is associated with menstrual disturbances such as PMS or dysmenorrhea (45). “Disturbed sleep” also seemed to be one of the most reported symptoms in this study (73.3% of athletes). This finding is in line with the systematic review of Taim et al. where insomnia/hypersomnia is one of the most common affective symptom with a prevalence of 53.3% (46.3–60.3%) in athletes (5). Sleep is important for recovery from the waking period through repair processes and regeneration (46). Therefore, sleep influences sporting performance in direct and indirect ways, e.g., by impacting symptom prevalence, neurophysiology, and cognitive function (47, 48).

In this study, we did not validate menstrual cycle phases, but instead, we performed a calculated approximation to categorize cycle phases in order to put symptoms into a temporal context. In agreement with current research (3, 6, 8, 10), in this study 15 out of 18 symptoms were more common during “menstruation” (0.79 symptoms per day) compared to the calculated “follicular phase” (0.08 symptoms per day), “luteal phase” (0.09 symptoms per day), and “late luteal phase” (0.00 symptoms per day). Having more symptoms during menstruation could mean a greater influence on training, days missing training, and perceived performance compared to other phases with less reported symptoms (49).

In this study, cycle length was calculated by tracking menstrual bleeding of athletes during 4‒20 months. A study comparing athletes with non-athletes found a higher incidence of irregular periods and heavy menstrual bleeding in athletes but no significant differences in the gynecological health including, e.g., pelvic pain (11). Although cycle lengths can vary in a normal menstrual cycle, a continuously prolonged menstrual cycle (e.g., longer than 35 days) is called “oligomenorrhea” (27, 50). Compared to a non-exercising population, athletes are more likely to have irregular cycles or menstrual cycle abnormalities (5, 51). Findings of this study illustrate the diversity of symptoms and menstrual cycle characteristics, e.g., cycle length. Out of 20 athletes, only 6 athletes had a natural cycle that was between 25 and 35 days with less than six days of variation during the measurement period. Six athletes reported a mean cycle length of over 35 days. In this study, the high mean values in the cycle length all appear to result from inconsistencies in the individual cycle lengths, e.g., VB07 reported no menstruation for 170 days at one point of the measurement but also had a longer period with cycle length between 25 and 35 days. VB15 was an exception with a consistent, long cycle in general. One athlete (VB18) had no complete cycle over the four-month measurement period at all. The athlete started the measurement at the onset of her menstruation but reported no second menstruation. Therefore, we were not able to calculate cycle length. Irregularities of cycle length in this study could be due to varying workloads during the season. In sports, oligomenorrhea and amenorrhea are often related to relative energy deficiency in sport (RED-S) resulting from a high training load and an insufficient dietary energy intake (21, 51). RED-S is associated with many health risks and with an decrease in performance (52, 53). However, it is possible that athletes do not feel any risk at the moment or are not aware of the consequences due to lack of adequate information, acceptance, and shame (25, 35, 49, 54). Fahrenholtz et al. show improvements of the regularity of menstrual cycle and also in LEA through an education-centered nutrition intervention in female athletes. These results were still visible in a 12 months follow-up (55).

Even though menstrual symptoms have consistently shown to be present in athletes (5), a study from McHaffie et al. (54) shows that coaches are not able to sufficiently notice the athletes' MCS, even though coaches report, that they feel athletes would talk to them if there was a need (54). Communication about menstruation between coaches and athletes still seem to be insufficient (8, 22–25, 56). A reason could be, that some athletes think that their coaches are not sufficiently informed (24) and that they would not be capable of helping them (25, 54). Interestingly, even though most coaches are male (57), studies have shown, that athletes tend to prefer communicating with female coaches (9, 23, 56). Additionally, some studies showed that some athletes feel there is no discrete or easy way to start communication (8). Taim et al. propose to foster a safe space by implementing a structured way of communication (58). Therefore, tracking could be used to get an idea about an athlete's well-being, open communication, and to suggest strategies to improve the athlete's well-being and training capacity. Even though some athletes do not feel that they need more specific support (54), talking about the menstrual cycle and forming an open environment seems to be generally appreciated (25). As this study shows, tracking symptoms can be kept easy and could help to obtain an overview of an athlete's personal experience. Some athletes are influenced more by their MCS than others and, thus, may need more support (54). Using the MSi score (4), medical support staff can get a good general view of their athletes' needs and well-being. The method is easy to integrate into a daily sport's routine and results regarding symptom severity and frequency has been shown to correlate with the risk of missing training or competition (4). By continuously tracking symptoms and evaluating the MSi regularly, symptom management can be controlled and adapted.

This study focused on the variations and fluctuations that a practitioner must take into consideration in a team. This study only included tracking MCS and menstrual bleeding (or withdrawal bleeding for HC users, respectively) for one club. The characteristics of this team may not be indicative of other teams and results may not be applicable for other sport teams. Future studies should investigate multiple teams to facilitate comparative analysis. Additionally, we did not include any influencing factors that may occur in elite sports, e.g., training load or traveling. It would be interesting to explore the factors that might influence cycle irregularities, but also the type or severity of a symptom. This also involves conducting a more comprehensive investigation of athletes using HC. As Bergström et al. showed, some coaches do not know, where to start adapting training strategies to menstrual cycle (22). Getting an overview could be a start and further information could help to implement changes in an athlete's lifestyle to improve their well-being and performance. In addition, tracking menstrual bleeding is not sufficient to measure the exact menstrual phases for the individual athletes. To determine menstrual cycle phases with certainty, it is inevitable to measure hormone concentrations and to determine ovulation (27), which were not conducted in this study. Julian and Sargent recommend using hormonal measurements, temperature measuring, and ovulation kits in research. But due to restricted resources and due to practicability they recommend using diaries and tracking systems in real life training settings (16). However, calculating menstrual cycle phases do not acknowledge the challenges of accurately determine phases. Nevertheless, this study aimed to test and describe a real life setting where hormonal testing is not available. Tracking methods are more accessible, non-invasive, and show a better acceptance among athletes (16).

## 5 Conclusion

In summary, the MCS and their frequency were found to vary widely between the athletes of an elite female volleyball team. Some of the athletes rarely recognized any symptoms while others suffered a high number of symptoms during their menstrual cycle. In addition, the cycle length varied between the athletes and within the individual athlete even in athletes using HC. By tracking menstrual cycle, athletes can develop personalized healthcare plans to enhance their well-being and comfort. Additionally, coaches can adjust training schedule according to an athletés menstrual cycle to optimize recovery and to support an athlete's well-being, including monitoring their health. Tracking menstrual cycle may also facilitate communication between athletes and sports staff by identifying individual needs for support. Future research should investigate individual solutions for every team athlete, such as management strategies regarding MCS and strategies for improving menstrual health. Therefore, more information is needed about parameters influencing menstrual cycles such as adequate regeneration, load decrement, nutrition, stress management and the use of suitable contraceptives, if desired. Tracking MCS and quantifying them by calculating an MSi score could offer a practical solution for sport practitioners to better understand menstrual cycles of their athletes.

## Data Availability

The original data can be made available on reasonable request. Requests to access the datasets should be directed to Andrea Roffler, roffler@sport.uni-frankfurt.de.
